# A cross sectional survey on UK older adult’s attitudes to ageing, dementia and positive psychology attributes

**DOI:** 10.1186/s12877-022-03539-w

**Published:** 2022-11-05

**Authors:** Madeleine Thelu, Bobbie Webster, Katy Jones, Martin Orrell

**Affiliations:** 1 School of Medicine, University of Nottingham, Nottingham, UK; 2 Academic Unit of Mental Health and Clinical Neuroscience, School of Medicine, University of Nottingham, Nottingham, UK

**Keywords:** Ageing, Dementia, Attitudes, Belief in a just world, Sense of coherence, Positive psychology, Stigma

## Abstract

**Background:**

With an increasingly ageing population worldwide, the predominant attitude towards ageing is still negative. Negative stereotypes have detrimental effects on individuals’ physical and mental health. Evidence is required about factors that may predict and change these views. This study aimed to investigate if an older person’s attitude towards dementia, their belief in a just world and sense of coherence is associated with their attitudes to ageing.

**Methods:**

A 25-min online survey was completed by 2,675 participants aged 50 or over who were current residents of the United Kingdom (UK). Questions included demographics, overall health, dementia carer, dementia relative status and retirement status. Standardised scales used were the Attitudes to Ageing Questionnaire (AAQ), Dementia Attitudes Scale (DAS), Just World Scale (JWS) and Sense of Coherence Scale-13 (SOC). Data was analysed with descriptive, two-tailed bivariate Pearson’s correlations, simple, and hierarchical regression analyses.

**Results:**

Attitudes to dementia, just world beliefs, and sense of coherence were all significantly positively correlated with AAQ-Total, with SOC sub-scale “Meaningfulness” showing the strongest correlation. In a hierarchical regression model, higher scores on SOC-Meaningfulness, DAS-Total and belief in a just world for oneself all predicted more positive attitudes to ageing.

**Conclusions:**

The more positive an individual’s attitude to dementia and the stronger they hold the belief that the world is just and coherent, the more likely they are to display positive attitudes to ageing. This initial evidence helps create a greater understanding of the factors that drive attitudes and stigma and may have implications for public health messaging.

## Background

Worldwide, the population is increasingly ageing [1], creating multiple challenges for society and healthcare systems due to issues such as neurodegeneration and multimorbidity requiring more complex care needs [2, 3]. Although positive stereotypes of older adults such as “generous” and “wise” exist [4], attitudes towards ageing in Western societies remain predominantly negative, with ageing seen as a time of decreased physical function, frailty, and being a burden on society [5–8]. Approximately 80% of adults over the age of 60 report experiencing ageism (defined as prejudice or discrimination towards an individual or group of people on the basis of their age) [9]. Common examples include jokes made at a person’s expense, or being treated with disrespect due to their age [10]. Negative stereotypes of ageing have been related to an increase in depressive symptoms whilst also having a negative influence on memory in older people [11, 12]. Weiss [13] found priming older adults with negative ageing stereotypes (e.g. being inactive) through a multiple-choice quiz led to an increase in systolic blood pressure reactivity, suggesting that negative views towards ageing may increase the risk of individuals developing cardiovascular problems. On the other hand, holding positive attitudes towards ageing and retirement have been linked to better emotional functioning and psychological health in older people [14, 15]. Focusing on what drives negative attitudes to ageing and the characteristics of those who hold them is necessary to improve older adults’ wellbeing and health [16]. Challenging negative views about ageing can be achieved through education and public health policies [8, 17, 18]. However, we need more information about what drives positive or negative attitudes to ageing.

Women generally held more positive views towards the physical changes of ageing [14], but saw it as a time of greater psychosocial loss, while men viewed ageing as a time of considerable physical loss [19]. Those with a greater degree of disability had more negative views of ageing and having a diagnosis of dementia was more predictive of having a negative attitude to ageing [14, 20]. Negative attitudes to ageing have also been linked to the development of brain changes which are associated with the development of Alzheimer’s Disease. From MRI scans, Levy et al. [21] found individuals with more negative attitudes to ageing had a greater decrease in hippocampal volume, with this part of the brain being crucial for memory function. There is less work about the impact of caring for a person with dementia on attitudes to ageing. One in three individuals in the UK will care for someone with dementia during their lifetime [22] and many people who care for individuals with dementia experience a negative effect on their wellbeing, known as “Carer Burden” [23]. One piece of work exploring attitudes to dementia and ageing found a more positive view of ageing and being a care-giver for someone with dementia was predictive of having more positive attitudes to dementia [24].

An individual factor that may influence attitudes to ageing is retirement. Retirement research has progressed from the notion of a discrete event in life to an adaptive process to maintain psychological wellbeing, whilst also adapting to the changes that retirement brings [25]. Almost half of working individuals expect retirement to be a time of uncertainty, whilst just below 30% see it as bringing hope of change [26]. In the longitudinal study ‘Like a High Wave: Adjustment to Retirement’, common views about retiring range from “I’m confused” to “having fun” and “there’s life after retirement” ([26] p. 231–232). A content analysis conducted one year after retirement revealed that most people had reassessed their previous expectations and found retiring to be a better experience than expected [26]. Individuals who were retired showed better psychological health measures compared to working individuals, with retirees having lower stress levels and a greater quality of life [15]. Conversely, research has also shown that retirees report a decreased level of happiness [27]. Retirement plays an important part in the experience of ageing, but it is unclear how it is associated with attitudes to ageing.

### Positive Psychology Constructs: the role of belief in a just world and sense of coherence on health and wellbeing

Positive psychology covers the processes and conditions that contribute towards the flourishing of people and what makes life worth living, looking at concepts such as wellbeing [28]. Wellbeing in later life has been revealed to be affected by individuals attitudes to ageing [29], however little is known regarding positive psychology in older adults and attitudes to ageing. One positive psychology model for wellbeing is Seligman's PERMA model, consisting of: Positive emotion, Engagement, Relationships, Meaning and Accomplishments [30]. In the context of the model, meaning involves one’s sense of purpose, judgements surrounding purpose and the sense created by experiences made from oneself, the relationships they share with others and the world [30–32]. Two positive psychology constructs that are relevant to the “Meaning” component of this model are Belief in a Just World and Sense of Coherence. In short, the ‘Belief in a Just World’ (BJW) hypothesis is an adaptive function built on the belief that the world is a just place, with each person’s actions having morally fair consequences, enabling individuals to cope with the worlds injustices [33]. The hypothesis includes BJW for oneself (BJW-self) and for others (BJW-others) [34]. Personal, but not general, BJW showed a positive relationship with self-esteem and life satisfaction [35] and better subjective wellbeing when other variables, such as self-reported health, were controlled [36]. Also, BJW-others was found to be associated with prejudice towards older adults, whilst only a higher general belief in a just world predicted psychological resilience [37, 38]. A study of 354 Lebanese nursing home residents found that those who scored higher on just world beliefs had lower depression scores, suggesting that it may act as a coping mechanism [39].

Sense of Coherence (SOC) examines how people use resources available to them to create a sense of control in life, therefore creating a coping capacity to deal with everyday stressors [40] and is associated with better quality of life [41]. SOC includes three components: Comprehensibility, Manageability, and Meaningfulness [42]. SOC has also been found to be a significant predictor of adjustment to ageing and can also act as a protective factor against experiencing caregiver burden [43, 44]. Higher SOC is associated with an increased frequency of physical exercise, a higher subjective rating of health, and decreased life stress [45–47]. Research has not explored how these constructs are related to attitudes to ageing.

### Aims

With previous research focusing on ageism and its association with health, the aim of the study was to investigate the interrelationships between attitudes to ageing, attitudes to dementia and positive psychology constructs belief in a just world and sense of coherence. The study also aimed to fill the gap in the work to explore caregiving and how this is linked to attitudes to ageing, as well as the link to retirement. It was hypothesised that attitudes towards dementia, belief in a just world and sense of coherence would be associated with individuals’ attitudes to ageing. It was also predicted that there would be relationships between demographic variables (age, highest educational attainment, UK location, ethnicity, health, and retirement status) and attitudes to ageing.

## Methods

### Study design

The study was a quantitative, online, cross-sectional survey available on JISC (a higher education online survey platform) and promoted on Join Dementia Research (JDR), an online self-registration UK resource which enables any individual to volunteer in dementia research. Around 52,000 people are registered with JDR, and from this about one in seven have a diagnosis of dementia. In total, 38,983 individuals met the study's eligibility requirements. Given the sensitive nature of some on the survey questions, details of supportive resources were provided to all participants. Ethical approval was granted by the Division of Psychiatry and Applied Psychology Research Ethics Committee at the University of Nottingham (Reference Number 2831). The sample size calculated to determine a significant correlation was 160 participants (bivariate normal model, Pearson’s R). This was calculated using *p* < 0.05 as a significance level, a power of 0.8, and a medium effect size of 0.3.

### Participants

Participants were UK residents, aged 50 years or above, who had signed up to JDR, and were able to read and understand the participant information sheet. Individuals could participate regardless of if they did or did not have a diagnosis of dementia as well as whether they were or were not retired. There were no other restrictions on participation.

### Measures

Demographic data collected included: age, education, UK region, ethnicity, general health, and retirement status. Mobility, hearing, and vision issues were assessed by “Yes” or “No”. Participants were asked to describe their overall health on a five-point Likert scale from “Very Poor” to “Very Good”. They were asked if they were a dementia carer or were related to someone with dementia (Yes/No). Participants were asked if they described themselves as retired (Yes/No). If “Yes”, they were asked to select as many reasons as to why they retired from a list of 13 options. These options were chosen as they were the most common reasons for retirement [48, 49]. Options for “Other reason” and a free text box was also provided.

### Attitudes to ageing

The Attitudes to Ageing Questionnaire (AAQ, [50]) measured: “Psychological Growth” (the degree to which participants view ageing as an opportunity for positive change), “Physical Change” (views towards the physicality’s of ageing and how they relate to beneficial health behaviours) and “Psychosocial Loss” (views towards ageing being a period of negative change). Each domain included eight statements that were assessed using a five-point Likert scale which ranged from “strongly agree” to “strongly disagree”, creating 24 items in total. A maximum score of 40 was available for each subscale and the overall maximum possible score was 120. The greater the overall score, the more positive the attitude towards ageing [50]. The AAQ has been found to reliably assess older adults’ attitudes to ageing [51] and is aimed to be used with older adults [14].

### Attitudes to dementia

The Dementia Attitudes Scale (DAS, [52]) measured “Dementia Knowledge” (how much participants believe individuals with dementia have a decreased ability to carry out seemingly everyday tasks), and “Social Comfort” (how relaxed participants feel in the presence of someone with a dementia diagnosis). Each subscale had 10 items, giving a total of 20 statements overall. Each statement was rated from “strongly agree” to “strongly disagree” on a seven-point Likert scale, giving each subscale a maximum possible score of 70 and a total maximum possible score of 140. Higher scores indicated a more positive attitude towards dementia. The scale has been shown to have good internal reliability and convergent validity with a Cronbach’s alpha score between 0.83 – 0.85 and having a strong correlation to attitudes to ageing and disability scales [52].

### Belief in a just world

The Just World Scale (JWS, [53]) measured participants just world beliefs. The scale separated procedural justice beliefs (equal procedures and treatments) from distributive justice (fair outcomes and beliefs about a fair world which relate to themselves (JWS-Self) and other people (JWS-Others)). All four subscales were found to have strong internal consistency and the higher order components of the scale were found to be significantly correlated with each other, with the JWS-Self and JWS-Other model showing the strongest significant positive correlation (*r* = 0.81, *p* < 0.001) [53]. There were 16 statements with each item being rated from “strongly agree” to “strongly disagree” on a seven-point Likert scale. A higher score indicated higher just world beliefs and the maximum score available was 112. Each subscale (JWS-self or JWS-others) had a maximum score of 56 [53].

### Sense of coherence

The Sense of Coherence Scale-13 (SOC-13, [54]) measured participants coping capacity to deal with everyday stressors. The three subscales measured: “Comprehensibility” (“Do you have very mixed-up feelings and ideas?”), “Manageability” (“Has it happened that people whom you counted on disappointed you?”) and “Meaningfulness” (“How often do you have the feeling that there’s little meaning in the things you do in your daily life?”). There were 13 statements ranked on a seven-point Likert scale. The maximum score was 91, with higher scores indicating a stronger sense of coherence [54]. Eriksson and Lindström [55] showed the SOC-13 to be as valid and reliable as the SOC-29 [56]. The SOC-13 showed high internal validity with a Cronbach’s alpha of 0.84 [57].

### Procedure

The survey link was shared on JDR. Those signed up to the platform were alerted or “matched” to the study and sent the link to take part. Participants provided online informed consent. Participants were aware they could withdraw their consent at any time (up until submitting their final data) by clicking out of the survey. The survey took approximately 25 min to complete. Participants also had the option to enter themselves into a prize draw to win one of two Amazon vouchers (worth £100 and £50). The survey was available from 05/11/2021 to 15/11/2021.

### Statistical analysis

Statistical analysis was conducted using Statistical Package for the Social Sciences (SPSS), Version 28. The purpose of the analysis was to look at the relationships between attitudes to ageing and the other variables and to assess whether the other variables predicted attitudes to ageing. For all analysis, *p* < 0.05 was used as the significance level. Descriptive analysis included frequencies, means and standard deviations. Between group analysis was performed to examine AAQ mean scores in relation to the demographic variables. Mean scores were calculated for AAQ-Total and each of the AAQ domains and these were analysed in relation to DAS, JWS and SOC-13 scores and overall health to examine for significance in two-tailed, bivariate Pearson’s correlation tests (since they all met the assumptions required: continuous variables, linearity, no significant outliers, and normality). The remaining categorical demographic variables (mobility, hearing and vision issues, dementia carer and relative status and retirement status) were tested to confirm they met the assumptions required for independent samples T-Tests (independence, no significant outliers, normality, and homogeneity of variances). These were then compared on AAQ-Total scores using independent samples T-Tests. Scales that were significantly correlated were tested to confirm they met the assumptions required for linear regression (linearity, homoscedasticity, independence, and normality). These were assessed using individual simple linear regression models. AAQ-Total was the dependent variable, and the corresponding scale/subscale was the independent variable.

A hierarchical regression model was created to investigate the effects of demographic variables on the relationship between AAQ-Total scores. The variables used were tested to confirm they met the assumptions required for hierarchical regression (linearity, homoscedasticity, independence, normality, and multicollinearity). Demographic data was recoded as follows: educational level into two levels (lower than undergraduate degree, undergraduate degree or above) and overall health into three levels (Poor, Satisfactory and Good). The “Enter” method was used and variable input order was: (a) age, (b) education, (c) retirement status, (d) mobility, hearing, and vision issues, (e) overall health, (f) dementia carer and relative, (g) SOC-Meaningfulness, (h) DAS-Total and (i) JWS-Self.

## Results

### Descriptive results and mean scores

A total of 2,676 people clicked on the survey link and 2,675 people went on to complete the survey (response rate of 99.9%). Individuals may not have clicked the survey link due to them being eligible for too many studies or deciding they did not want to participate. Also, they might not have completed the survey as they no longer wanted to participate. No question was unanswered in the survey. The mean age was 66.0 (*SD* = 8.1 years) and most participants had an undergraduate degree or higher (59.7%) (Table 1). Most participants were white (98.2%) and had no problems with mobility (90.4%), hearing (78.4%) or vision (63.9%). Most described their overall health as good (45.9%) or very good (29.6%). The majority (57.5%) had not been a dementia carer, but over half were related to someone with dementia (52.7%). Overall mean score for attitudes to ageing was 87.8 out of a possible 120. Total AAQ scores organised by demographic groups are presented in Table 1.

**Table 1:** Table summarising demographic information and between group analysis results for AAQ-Total

Variable	Frequency *n (%)*	AAQ-Total Mean (*SD*)
Highest Educational Level		
None	33 (1.2)	87.4 (10.6)
GSCE or Equivalent	248 (9.3)	
AS Level	6 (0.2)	
A Level or Equivalent	231 (8.6)	
Vocational Training Certificate or Diploma	498 (18.6)	
Undergraduate Degree	863 (32.3)	88.1 (9.79)
Postgraduate Degree	732 (27.4)	
Prefer not to say	6 (0.2)	-
Other	58 (2.2)	-
Ethnicity		
White	2626 (98.2)	87.8 (10.1)
Mixed or Multiple Ethnic Groups	23 (0.9)	88.0 (7.3)
Asian or Asian British	10 (0.4)	86.4 (11.4)
Black, African or Caribbean	4 (0.1)	92.3 (10.9)
Other Ethnic Groups	2 (0.1)	90.0 (12.7)
Prefer not to say	10 (0.4)	81.3 (8.6)
Any Self-Reported Problems with Mobility		
Yes	256 (9.6)	80.4 (11.1)
No	2419 (90.4)	88.6 (9.7)
Any Self-Reported Problems with Hearing		
Yes	578 (21.6)	85.5 (10.2)
No	2097 (78.4)	88.5 (10.0)
Any Self-Reported Problems with Vision		
Yes	965 (36.1)	86.5 (10.3)
No	1710 (63.9)	88.5 (9.2)
Self-Reported Description of Overall Health		
Very Poor	3 (0.1)	73.7 (9.3)
Poor	75 (2.8)	74.3 (10.2)
Satisfactory	578 (21.6)	81.2 (9.6)
Good	1227 (45.9)	88.2 (8.5)
Very Good	792 (29.6)	93.4 (8.6)
Dementia Carer History		
Yes	1138 (42.5)	87.8 (10.2)
No	1537 (57.5)	87.8 (10.0)
Relative with Dementia		
Yes	1409 (52.7)	88.0 (10.3)
No	1266 (47.3)	87.6 (9.9)
Retirement Status		
Yes	1838 (68.7)	87.5 (10.0)
No	837 (31.3)	88.6 (10.2)

Due to the effects of rounding, not all percentages total to 100. AAQ = Attitudes to Ageing Questionnaire

A total of 68.7% of the sample were retired with main reasons including being able to afford retirement (15.1%), retiring to enjoy life (12.9%), and reaching retirement age (12.5%). Common themes in the “Other” category included redundancy/early retirement (*n* = 59), volunteering (*n* = 21), and relocation (*n* = 9) (See Table 2).

**Table 2:** Table summarising retirement data

Variable	Frequency *n (%)*
Retirement Status	
Yes	1838 (68.7)
No	837 (31.3)
Reasons for Retirement	
Can Afford to Retire	904 (15.1)
To Enjoy Life	773 (12.9)
Reached Retirement Age	752 (12.5)
Wish to Retire	752 (12.5)
More Time for Leisure	719 (12.0)
To Spend Time with Loved Ones	530 (8.8)
To Travel	514 (8.6)
Dissatisfied with Job	303 (5.1)
Caring for Family Member	215 (3.6)
Other	187 (3.1)
Poor Health	139 (2.3)
Unforeseen Circumstances	121 (2.0)
Poor Health of a Relative or Friend	87 (1.5)
Caring for Friend	2 (0.0)

Due to the effects of rounding, not all percentages total to 100

For each standardised scale and subscale, the means, standard deviations, minimum and maximum scores, and skew values are presented in Table 3. The skew values for AAQ-Total, DAS-Total and JWS-Total show the data follows a normal distribution. SOC-Total shows a moderate negative skew.

**Table 3:** Table summarising the means, standard deviations, ranges and skew for all scales and subscales

Variable	Mean (*SD*)	Minimum and Maximum Score	Skew
Attitudes to Ageing Questionnaire			
AAQ-Total	87.8 (10.1)	44—117	-0.38
AAQ-Psychosocial Loss	28.4 (5.0)	8—40	-0.40
AAQ-Physical Change	30.4 (4.7)	12—40	-0.44
AAQ-Psychological Growth	29.0 (3.4)	17—40	-0.15
Dementia Attitudes Scale			
DAS-Total	107.8 (15.0)	56—140	-0.05
DAS-Social Comfort	49.1 (10.3)	14—70	-0.09
DAS-Dementia Knowledge	58.5 (6.7)	28—70	-0.47
Just World Scale			
JWS-Total	65.7 (14.7)	16—112	-0.27
JWS-Others	28.3 (8.5)	8—56	-0.60
JWS-Self	37.4 (8.1)	8—56	-0.10
Sense of Coherence Scale-13			
SOC-Total	66.2 (12.2)	15—91	-0.54
SOC-Comprehensibility	24.4 (5.4)	5—35	-0.45
SOC-Manageability	20.2 (4.4)	4—28	-0.54
SOC-Meaningfulness	21.6 (4.2)	4—28	-0.70

*AAQ* Attitudes to Ageing Questionnaire, *DAS* Dementia Attitudes Scale, *JWS* Just World Scale, *SOC* Sense of Coherence Scale-13. Maximum possible score for AAQ-Total of 120 and for each AAQ subscale of 40. Maximum possible score for DAS-Total of 140 and for each DAS subscale of 70. Maximum possible score for JWS-Total of 112 and for each JWS subscale of 56. Maximum possible score for SOC-Total of 91, for SOC-Comprehensibility of 35 and for SOC-Manageability and SOC-Meaningfulness each a maximum possible score of 28

More positive attitudes to ageing were significantly correlated with more positive attitudes to dementia, stronger just world beliefs, and a greater sense of coherence (Table 4). The correlations with the strongest relationships were found to be between AAQ and SOC (and their subscales) and between AAQ and overall health. Educational level was weakly correlated with AAQ-Psychological Loss (See Table 4).

**Table 4:** Two-tailed, bivariate Pearson Correlation Coefficients for correlations between AAQ and DAS, JWS and SOC scales

	**AAQ-Total** **(95% CI)**	**AAQ-Psychosocial Loss** **(95% CI)**	**AAQ-Physical Change** **(95% CI)**	**AAQ-Psychological Growth** **(95% CI)**
DAS-Total	0.275** (0.240–0.310)	0.228** (0.192–0.263)	0.168** (0.131–0.204)	0.248** (0.213–0.284)
DAS-Social Comfort	0.234** (0.198–0.270)	0.222** (0.186–0.258)	0.139** (0.102–0.176)	0.176** (0.139–0.212)
DAS-Dementia Knowledge	0.254** (0.218–0.289)	0.167** (0.130–0.204)	0.161** (0.123–0.197)	0.285** (0.250–0.320)
JWS-Total	0.268** (0.233–0.303)	0.193** (0.156–0.229)	0.232** (0.196–0.268)	0.192** (0.156–0.229)
JWS-Self	0.323** (0.288–0.356)	0.253** (0.217–0.288)	0.280** (0.245–0.314)	0.200** (0.163–0.236)
JWS-Others	0.157** (0.119–0.193)	0.092** (0.055–0.130)	0.135** (0.097–0.172)	0.143** (0.105–0.179)
SOC-Total	0.510** (0.482–0.538)	0.524** (0.496–0.551)	0.394** (0.362–0.426)	0.199** (0.163–0.235)
SOC-Comprehensibility	0.387** (0.355–0.419)	0.405** (0.373–0.473)	0.307** (0.272–0.341)	0.130** (0.092–0.167)
SOC-Manageability	0.411** (0.379–0.442)	0.443** (0.412–0.473)	0.331** (0.297–0.365)	0.111** (0.073–0.148)
SOC-Meaningfulness	0.557** (0.530–0.582)	0.540** (0.513–0.566)	0.406** (0.374–0.437)	0.297** (0.262–0.331)
Highest Educational Level	0.032 (-0.006–0.069)	0.045* (0.007–0.082)	0.035 (-0.003–0.073)	-0.280 (-0.059–0.017)
Overall Health	-0.483** (-0.511–0.453)	-0.369** (-0.401–0.336)	-0.548** (-0.574–0.521)	-0.137** (-0.174–0.99)

^*^ = *p* < 0.05, ** = *p* < 0.01, *AAQ* Attitudes to Ageing Questionnaire, *DAS* Dementia Attitudes Scale, *JWS* Just World Scale, *SOC* Sense of Coherence Scale-13

Independent-sample T-Tests were conducted to assess for significant differences in AAQ-Total scores between different demographic groups (mobility, hearing and vision issues, dementia carer and relative and retirement status) (Table 5). Participants with no mobility issues had a higher mean AAQ-Total score (*M* = 88.6, *SE* = 0.197) than those with mobility issues (*M* = 80.4, *SE* = 0.696) and this difference was significant (*t*(2673) = 12.776, *p* < 0.001) with a large effect size (*d* = 0.840). Participants who were not retired (*M* = 88.6, *SE* = 0.354) had more positive attitudes to ageing compared to those who were retired (*M* = 87.5, *SE* = 0.234) and this difference was significant (*t*(2673) = 2.571, *p* < 0.01) with a small effect size (*d* = 0.107).

**Table 5:** T-test results (t) and significance levels of the mean differences between AAQ-Total and demographic variables

		***t- statistic***	**Mean diff**	***P* v alue**	**Cohen’s d (*d*)**	**95% CI**		**SE**
						**Lower**	**Upper**	**Mean**
**AAQ-Total**	**Mobility Issues**	12.776	8.242*	< .001	0.840	6.977	9.507	0.197
	**Hearing Issues**	6.356	2.997*	< .001	0.299	2.072	3.921	0.218
	**Vision Issues**	4.905	1.988*	< .001	0.197	1.193	2.783	0.240
	**Dementia Carer**	0.119	0.047	.905	0.005	-0.728	0.823	0.255
	**Dementia Relative**	-1.222	-0.478	.222	-0.047	-1.246	0.289	0.277
	**Retirement Status**	2.671	1.083*	.010	0.107	0.257	1.909	0.354

^*^ = *p* < 0.01, *AAQ* Attitudes to Ageing Questionnaire

### Linear and Hierarchical Regression Analysis for AAQ-Total Scores

Linear regression calculations were conducted to predict AAQ-Total score (See Table 6). DAS-Total accounted for the largest amount of variance in AAQ-Total (*R*^*2*^ = 0.076) compared to DAS-Social Comfort (*R*^*2*^ = 0.055) and DAS-Dementia Knowledge (*R*^*2*^ = 0.064). DAS-Total was shown to be a significant predictor of AAQ-Total, where for every unit increase in DAS-Total, an increase of 0.185 was seen in the AAQ-Total score (*β* = 0.185 (*p* < 0.001, 95% CI [0.161, 0.210]). Also, the largest increase in AAQ-Total in relation to a unit change in JWS scores was seen with JWS-Self as a predictor (*β* = 0.402, *p* < 0.001, 95% CI [0.357, 0.446]) and JWS-Self alone explained 10.4% of the variance observed in AAQ-Total, compared to JWS-Total explaining 7.2% (*R*^*2*^ = 0.072) and JWS-Others explaining 2.4% (*R*^*2*^ = 0.024). Overall, SOC-Meaningfulness as a predictor explained the greatest variance in AAQ-Total scores, where *R*^*2*^ = 0.310, compared to SOC-Total (*R*^*2*^ = 0.261). SOC-Meaningfulness was the largest significant predictor for AAQ-Total scores overall, where AAQ-Total = 58.752 + 1.345*(SOC-Meaningfulness) (β = 1.345, *p* < 0.001, 95% CI [1.269, 1.421]).

**Table 6:** Linear regression results for DAS, JWS and SOC scales as a predictor of AAQ-Total scores

		***R***^***2***^	***β* value**	**Constant**	**95% CI**	
					**Lower**	**Upper**
**AAQ- Total**	**DAS-Total**	0.076	0.185*	67.859	0.161	0.210
	**DAS-Social Comfort**	0.055	0.229*	76.577	0.193	0.265
	**DAS-Dementia Knowledge**	0.064	0.382*	65.373	0.327	0.437
	**JWS-Total**	0.072	0.184*	75.718	0.159	0.209
	**JWS-Self**	0.104	0.402*	72.798	0.357	0.446
	**JWS-Others**	0.024	0.186*	82.533	0.142	0.231
	**SOC-Total**	0.261	0.423*	59.815	0.396	0.450
	**SOC-Comprehensibility**	0.150	0.722*	70.217	0.657	0.787
	**SOC-Manageability**	0.169	0.954*	68.554	0.874	1.034
	**SOC-Meaningfulness**	0.310	1.345*	58.752	1.269	1.421

^*^ = *p* < 0.001, *AAQ* Attitudes to Ageing Questionnaire, *DAS* Dementia Attitudes Scale, *JWS* Just World Scale, *SOC* Sense of Coherence Scale-13

Hierarchical regression was conducted to see if and to what degree the variables used predicted AAQ-Total scores. DAS-Total, JWS-Self and SOC-Meaningfulness were the most significant predictors for AAQ-Total, thus were inputted into a hierarchical regression model (Table 7). The total R^2^ was 0.464. The demographic variables listed were controlled (ethnicity was excluded as 98.2% of participants were white). The following models significantly contributed towards the final model: Age (Model a) explained 2% of the variance in AAQ-Total scores: Mobility, hearing and vision issues (Model d) explained 6.6% of the variance: Overall health (Model e) explained 17.3% of the variance: SOC-Meaningfulness (Model g) explained the largest amount of variance, 18.5%, and finally both DAS-Total (Model h) and JWS-Self (Model i) explained 2.5 and 1.2% of the variance in AAQ-Total scores respectively. From the significant predictors of AAQ-Total, SOC-Meaningfulness had the highest beta value, followed by DAS-Total and then JWS-Self. Overall health had the highest negative beta value, followed by mobility, hearing and vision issues, with age having the lowest negative beta value (See Table 7).

**Table 7:** AAQ-Total hierarchical regression model produced for DAS-Total, JWS-Self and SOC-Meaningfulness when controlling for demographic variables

		**R**^**2**^** Change**	**F change**	**Sig F Change**	**Standardised Beta Coefficient**
**Model a**	**Age**	0.002	*F(*1,2673) = 4.156*	0.042	-0.039*
**Model b**	**Education**	0.001	*F*(1,2672) = 2.520	0.113	0.031
**Model c**	**Retirement Status**	0.001	*F*(1,2671) = 2.652	0.104	-0.039
**Model d**	**Mobility Issues**	0.066	*F*(3,2668) = 62.671***	< 0.001	-0.224*** -0.077*** -0.051**
	**Hearing Issues**				
	**Vision Issues**				
**Model e**	**Overall Health**	0.173	*F*(1,2667) = 610.002***	< 0.001	-0.452***
**Model f**	**Dementia Carer**	0.000	*F*(2,2665) = 0.757	0.469	-0.010 0.022
	**Dementia Relative**				
**Model g**	**SOC-Meaningfulness**	0.185	*F*(1,2664) = 859.356***	< 0.001	0.457***
**Model h**	**DAS-Total**	0.025	*F*(1,2663) = 119.127***	< 0.001	0.176***
**Model i**	**JWS-Self**	0.012	*F*(1,2662) = 58.282***	< 0.001	0.116***

^*^ = *p* < 0.05, ** = *p* < 0.01, *** = *p* < 0.001, *AAQ* Attitudes to Ageing Questionnaire, *DAS* Dementia Attitudes Scale, *JWS* Just World Scale, *SOC* Sense of Coherence Scale-13

## Discussion

This study is the first to examine the interrelationships between attitudes to ageing, dementia, retirement and positive psychology constructs, and establish strongest predictors of attitudes to ageing. Findings showed an individual’s attitudes to ageing were associated with their attitudes to dementia, belief in a just world, and overall sense of coherence. The strongest predictors of attitudes to ageing were SOC-Meaningfulness, then DAS-Total and JWS-Self respectively. Findings also showed an association between certain demographic factors (age, mobility, hearing and vision issues and overall health) and attitudes to ageing. Retirement status did not predict attitudes to ageing.

The mean AAQ score in this study was higher than in previous research, suggesting this sample overall held more overall positive attitudes to ageing. For example, this sample’s mean score of 87.8 was higher than 82.9 in 448 Turkish adults aged over 65 [58]. The sample in our study also showed more positive attitudes to dementia [59] compared to nursing students in Malta. This was somewhat unexpected as this survey was conducted during the COVID-19 pandemic, during which older adults in the UK were more likely to be isolated and told they were vulnerable, which could have influenced their attitudes. However, as recruitment was solely via JDR, the sample may have had a positive skew towards more positive attitudes to ageing and dementia as the participants were over 50 and predominantly white, well-educated individuals, who were interested in dementia research, and had internet access.

Overall, SOC-Meaningfulness had the strongest positive correlation with attitudes to ageing as well as it being the strongest significant predictor for overall attitudes to ageing. It could be suggested that individuals who consider their life to have emotional meaning when a problem occurs, perceive the world as an understandable place and can solve problems when they arise [42] are less likely to view ageing as a time of loss. They may also be less apprehensive about the uncertainties around ageing and feel better able to cope with the stressors of ageing. SOC overall has previously been found to be a significant predictor for better adjustment to ageing in adults aged over 60 years [43, 60]. Although, this research did not investigate participants attitudes to ageing specifically, thus our study adds further insight.

Results showed neither being a carer for a person with dementia, nor having a relative with dementia significantly predicted attitudes to ageing. Previous research has focused on whether having a diagnosis of dementia affects attitudes to ageing [20], or how attitudes to ageing affect dementia attitudes [24], which revealed positive attitudes to ageing as being a predictive factor for more positive dementia attitudes. However, research has not investigated whether dementia attitudes predict attitudes to ageing. Previous work in Germany showed a proportion of the population have a fear of dementia, and this fear was associated with being older, being female, and the view that developing dementia would ruin their life [61]. Fear of dementia may influence a person’s attitude to getting older. It is possible that this sample had less fear, thus a more positive attitude to ageing. Improving the general public’s knowledge of dementia through education and public health messaging may help reduce fear of dementia and ageing.

Belief in a just world for oneself was also shown to be a significant predictor for positive attitudes to ageing, implying that those who believed the world to be fair towards themselves [34] had a more positive view of ageing. This could be explained by those believing ageing well is a consequence of their positive actions, which enhance wellbeing and self-esteem. Research has found that individuals with greater personal just world beliefs showed increased emotional stability and greater satisfaction with life [34]. In contrast, Lipkus and Siegler [62] found people with high just world beliefs were more likely to show discriminatory views towards individuals because of their age.

Demographic, retirement, and health-related variables were explored in this study. Poor self-reported health was the strongest predictor of negative attitudes to ageing and mobility, hearing and vision issues, and increased age also predicted negative attitudes to ageing. Together, these variables explained a significant amount of variance in AAQ-Total scores. However, we were not able to include gender which could have influenced some variance in AAQ-Total score as consistent with previous research [14, 19]. The most common reasons for retirement were being able to afford to retire, wanting time to enjoy life, and reaching retirement age. A Swedish study suggested the most common reasons for retirement were health and work being too demanding [49]. However, cultural differences, as well as the study only including “push” factors (e.g. poor health) whilst excluding “pull” factors (e.g. more time to spend with loved ones) may explain the findings. Previous work found that retired individuals had lower level of happiness [27], which could explain why retired participants in our study held more negative views to ageing compared to non-retired participants. However, hierarchal regression analysis revealed retirement status was not an overall significant predictor for attitudes to ageing.

### Implications

These results have found further information surrounding potential causative factors which contribute towards attitudes to ageing. Sense of coherence could be utilised as a theoretical framework for health promotion as it focuses on using available resources to find and carry out solutions, enabling individuals to obtain control over the factors that determine their health [63]. Improved education and awareness about dementia, such as through dementia centred audio-visual novellas [17], can lead to more positive attitudes to ageing and since just world beliefs relating to oneself are associated with more positive attitudes to ageing, health messaging may focus on individuals, rather than focusing on another person.

### Strengths and limitations

Strengths include a convenient and easily accessible survey to enable a large sample size and as questions explore personal attitudes, social desirability bias was minimised due to the online format. Third, a robust exploration of different types of attitudes was undertaken. Also, there was no discrimination against individuals with dementia being able to be involved, as all the participants perspectives were valuable regardless of if they did or did not have a dementia diagnosis. Limitations include lack of generalisability and sampling bias, as only registered JDR users could participate in the online survey. Since dementia diagnosis data was not collected, sub-analysis regarding this was unable to be carried out. With regards to the participants attitudes towards dementia, there could have been more positive views due to a greater knowledge surrounding dementia, since participants were recruited from JDR. Conversely, there could have been more negative views as participants may have joined JDR if they were upset by either having a diagnosis of dementia, since they experience the challenges it brings, or if they sympathise with a family member with dementia. Future research in this area should include having a diagnosis of dementia in the analysis and utilise a range of sampling methods to recruit a sample with more ethnic and socioeconomic diversity, and a broader spectrum of age. In 2020, 54% of adults aged 75 and above were recent internet users [64], meaning a large proportion of older adults may not have been able to access the survey. This may explain why we recruited a smaller proportion of participants over 75. Gender data was not collected due to an omission on the online survey thus this variable could not be explored. Overall JDR gender ratio was around two-thirds women and one third men. Future research should include gender in analyses. In-depth data surrounding participants health was not collected due to the risk that an even longer questionnaire would decrease the response rate. Further studies could focus more on participants health and how different factors, e.g. age of onset of health problems, are linked to older adults’ attitudes to ageing. Also, randomisation of the order of presentation of measures was not carried out due to the format and platform of the study. Furthermore, alternative methods for the analysis exist, including multiple regression, for when there is not an underlying theory. Future research should aim to address these limitations. Analysis showed that, out of the total scores for the four scales used, only the total score for SOC showed a moderate negative skew. Finally, the study was cross-sectional which limits our understanding of how these constructs change over time. Attitudes to ageing may fluctuate through later life as health and other personal circumstances change.

## Conclusions

We found significant positive relationships between attitudes to ageing and dementia, belief in a just world and sense of coherence when demographics (such as age, health issues and retirement status) were controlled. A positive relationship was found between the meaningfulness sub-scale of sense of coherence, attitudes to dementia and a belief that the world is just for oneself and attitudes to ageing. Results illuminate new potential factors linked to attitudes to ageing which extends previous work focusing on health and demographic factors.

## Data Availability

The datasets generated and analysed during the current study are not publicly available due to being stored on a password protected University One Drive Account but are available from the corresponding author on reasonable request.

## Abbreviations

BJW: Belief in a Just World
SOC: Sense of Coherence
JDR: Join Dementia Research
UK: United Kingdom
AAQ: Attitudes to Ageing Questionnaire
DAS: Dementia Attitudes Scale
JWS: Just World Scale
SOC-13: Sense of Coherence Scale-13
SD: Standard Deviation
